# Huge Congenital Segmental Dilatation of the Sigmoid Colon in a Neonate: A “Rarity to Meet” and a “Challenge to Treat”

**DOI:** 10.1155/2016/9685307

**Published:** 2016-04-28

**Authors:** Margarita Kaiser, Christoph Castellani, Georg Singer, Robert Marterer, Manfred Ratschek, Holger Till

**Affiliations:** ^1^Department of Pediatric and Adolescent Surgery, Medical University of Graz, Auenbruggerplatz 34, 8036 Graz, Austria; ^2^Division of Pediatric Radiology, Medical University Graz, Auenbruggerplatz 34, 8036 Graz, Austria; ^3^Institute of Pathology, Medical University of Graz, Auenbruggerplatz 25, 8036 Graz, Austria

## Abstract

Only ten cases of neonatal congenital segmental dilatation (CSD) of the colon have been described so far. We present a full-term female newborn with trisomy 21, ventricular septal defect, and gross abdominal distension. Plain abdominal radiographs revealed a huge cystic lesion occupying the left hemiabdomen. Upon laparotomy on day 4 a CSD of the distal sigmoid and proximal rectum was confirmed and resected. The proximal colon was exteriorized and the distal part closed as a Hartmann pouch. Histology confirmed a huge segmental dilatation of the sigmoid without dysganglionosis or pseudodiverticula, but normal intestinal architecture. After correction of the ventricular septal defect a low rectal end-to-end anastomosis could be performed at an age of 5 months. The postoperative course was uneventful. CSD of the sigmoid colon is extremely “rare to meet” and a “challenge to treat” in the newborn period, but clinical awareness of this entity prompts pediatric surgical success.

## 1. Introduction

Congenital segmental dilatation (CSD) of the colon represents a rare congenital malformation most often diagnosed in children beyond the neonatal period. So far, only ten cases of neonatal colonic segmental dilatation have been described in the literature [1].

The main characteristics of CSD of the colon include a single large dilatation of the colon of variable length with an abundant serosal blood vasculature but lacking teniae coli, haustrations, and appendices epiploicae [1]. However, the proximal and distal parts of the colon display a normal caliber with an abrupt transition from the dilatation to the unaffected intestine. Interestingly, the left side of the colon is affected more often with an involvement of the sigmoid and rectosigmoid in almost half of the cases [2]. In some patients associations with other malformations such as malrotation, duodenal atresia, and facial defects have been found [1].

Herein, we report the findings and the operative management of a CSD of the colon in a neonate with trisomy 21 and ventricular septal defect.

## 2. Case Presentation

A full-term female newborn was spontaneously delivered with a birth weight of 2,600 g in a peripheral hospital. Additional findings were trisomy 21 combined with a ventricular septal defect. At day 1 the patient showed increasing abdominal distension. Clinically, the patient had a normal anal orifice and passed meconium within 14 h after birth.

**Figure 1:**
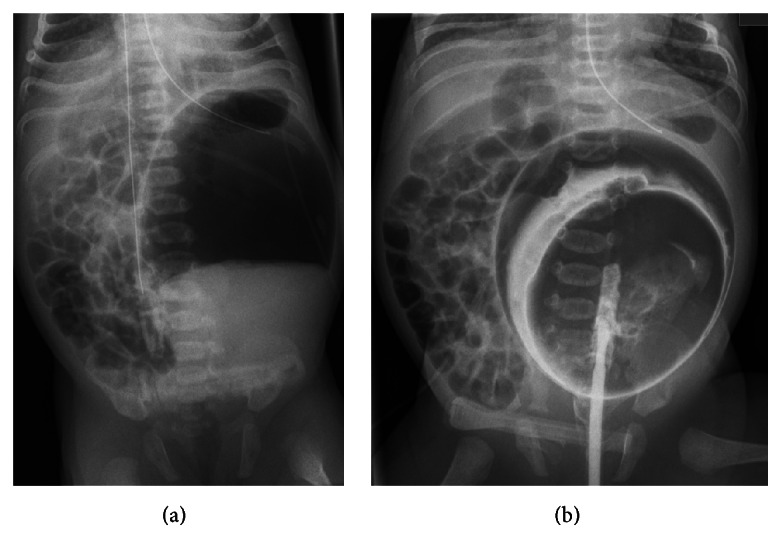
Upright anterior-posterior abdominal X-ray showing a big cystic lesion in the left hemiabdomen with complex contents (a); supine anterior-posterior abdominal X-ray after contrast enema (b).

The radiological work-up revealed a huge gas filled cystic lesion located in the left hemiabdomen and a consecutive displacement of the intestine (Figure 1(a)). The patient was referred to our center for further diagnosis and treatment on day 3 of life. Ultrasound revealed no signs of malformation of the inner female genitalia. A contrast enema showed contrast media in the cyst proving communication between the cyst and the intestine (Figure 1(b)). Several differential diagnoses were considered such as CSD, colonic duplication, and congenital pouch colon with high ARM and the patient was scheduled for surgery due to increasing intolerance of food.

**Figure 2:**
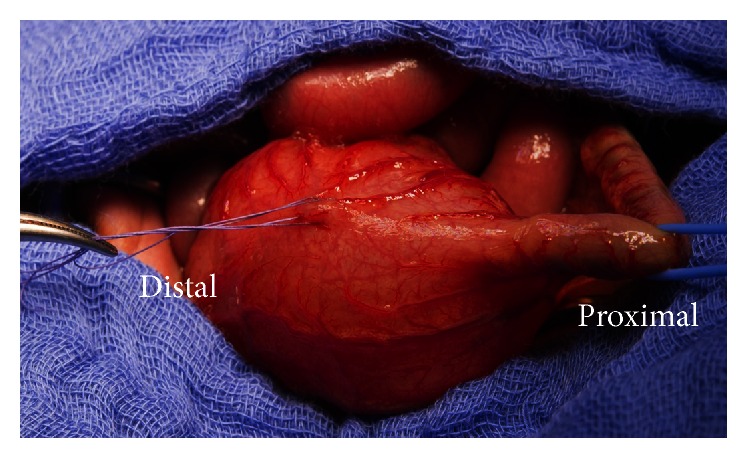
Macroscopic presentation at surgery with normal proximal colon (vessel loop) ending in a pouch measuring approximately 6 × 4.5 × 2 cm.

**Figure 3:**
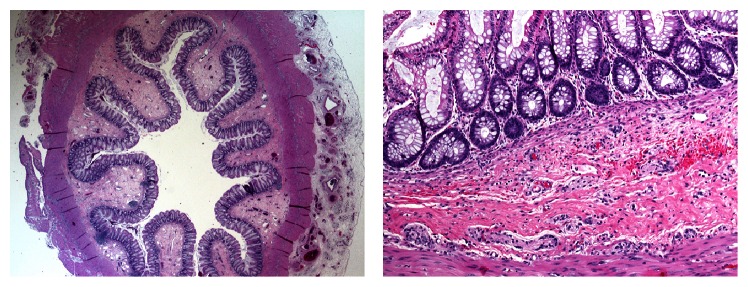
Histology of the CSD of the colon revealed normal architecture of the dilatation.

Upon laparotomy on day 4 of life a grossly dilated pouch-like segment of the sigmoid colon became evident reaching well beyond the peritoneal space of Douglas (Figure 2). No high fistula of an ARM could be identified. The dilated sigmoid was resected. For cardiac and surgical safety the distal rectum was closed and the normal sigmoid externalized as a temporary Hartman-colostomy. The histological work-up showed a grossly dilated colon with normal histological architecture and normal ganglion cells (Figure 3).

After correction of the ventricular septal defect by cardiac surgery closure of the colostomy was planned at an age of five months. Prior to surgery an anorectal manometry was performed showing inconspicuous results. Additionally, on rectal examinations relaxations were present. Therefore, Hirschsprung's disease was ruled out and a low rectal end-to-end anastomosis (below the peritoneal space of Douglas) could be performed. The postoperative course was uneventful. At the latest outpatient visit two months following the closure of the colostomy the patient was free of symptoms passing daily stool.

## 3. Discussion

In 1959, CSD of the colon was described by Swenson and Rathauser in a child for the first time [3]. Since then, the majority of the cases have been reported in infants and children and occasionally adults [4]. Only about ten cases with CSD of the colon have been reported in neonates so far.

To confirm the diagnosis of CSD of the colon Brawner and Shafer have reported the following five criteria: (I) lack of radiographically demonstrable motility of the dilated segment, (II) normally appearing and functioning colon both proximal and distal to the dilated segment, (III) absence of teniae coli in the dilated segment, (IV) normal ganglion cells, and (V) hypertrophy of circular and longitudinal muscle layers in the dilated segment [5]. These criteria are strikingly similar to another malformation termed congenital pouch colon. However, congenital pouch colon normally is associated with anorectal malformations where the dilated pouch ends either blindly or with a fistulous connection to the urogenital tract [1]. A plethora of different anatomical variants of the fistula have been reported [6]. Hence, the main difference between these two entities is the presence of a normal colon distally to the dilatation in CSD. Interestingly, there have also been reports describing the simultaneous occurrence of both congenital pouch colon and CSD of the colon [7].

Our patient fulfilled four out of the five Brawner criteria. While normal ganglion cells could be demonstrated histologically, no hypertrophy of the muscles layers was seen (compare Figure 3). This finding may be explained by the fact that hypertrophy of the muscle layers develops over time due to ongoing contraction in the affected part to overcome the chronic obstruction [2]. This is supported by reports describing hypertrophied muscle layers in late presenting and not in early presenting cases [2].

The exact pathogenesis of CSD of the colon is still a matter of debate. Available theories include intrauterine vascular incidents, defective organogenesis, strangulation of the intestine in the umbilical ring, neurogenic causes, and defective muscular development [1]. A wide variety of different associated malformations has been reported. These include duplex appendix, duplication of cecum, Meckel's diverticulum, meningomyelocele, hydrops of the gallbladder, colonic atresia, and facial defects [1]. Nevertheless, the association with trisomy 21 has only been reported once [8].

The common symptoms of children with CSD of the colon include abdominal distention, chronic constipation, and episodes of diarrhea and are therefore often treated in the line of suspected Hirschsprung's disease [4]. Hirschsprung's disease was ruled out by inconspicuous clinical and anorectal manometry findings in our patient. In neonatal cases, however, CSD of the colon may cause neonatal intestinal obstruction like in the presented patient. Rarely, it may present with peritonitis due to perforation or volvulus of the sigmoid colon [1].

The recommended treatment consists of resection of the affected segment with either end-to-end anastomosis or creation of a proximally located stoma [2, 9]. In critically ill patients, creation of an ileostomy without excision of the affected segment has been reported [9]. In more complex and unique cases, however, specifically tailored surgical strategies have to be applied. Ragavan and coworkers, for instance, have successfully treated a patient with segmental dilatation of almost the entire colon with tubularization of the segmental dilatation of colon with stoma formation as first stage followed by delayed anastomosis during a second stage [2].

In contrast to congenital pouch colon (CPC), intestinal duplication, and Hirschsprung's disease congenital dilatation of the sigmoid colon is extremely “rare to meet” and a “challenge to treat” in the newborn period, but clinical awareness of this entity prompts pediatric surgical success.
